# A highly integrated nonvolatile bidirectional RFET with low leakage current

**DOI:** 10.1016/j.heliyon.2023.e19298

**Published:** 2023-08-28

**Authors:** Xi Liu, Mengmeng Li, Shouqiang Zhang, Xiaoshi Jin

**Affiliations:** a School of Information Science and Engineering, Shenyang University of Technology, Shenyang 110870, China

**Keywords:** Reconfigurable FETs, Bidirectional, Nonvolatile, Low leakage

## Abstract

A highly integrated nonvolatile bidirectional reconfigurable FET controlled by a single gate (SGCN-BRFET) is proposed. The nonvolatile function, the bidirectional function and the reconfigurable function can be achieved at the same time. Instead of the independently powered program gate (PG) of BRFET, the program operation of the proposed SGCN-BRFET can be independently completed by the control gate (CG) itself through storing positive or negative charges in a floating program gate (FPG) formed on both source/drain sides. Thereafter, the interconnection can be simplified. The conduction type of the SGCN-BRFET is reconfigured by programming the FPG with different type of charges into the FPG. By optimizing the quantities of the stored charges, the FPG effective voltage can be changed to achieve higher forward current and lower leakage current. The physical mechanism of the proposed SGCN-RFET has been systematically analyzed. The device performance has been compared with BRFET. The influence of the amount of charge to the device performance has also been discussed in detail.

## 1 Introduction

At present, the most advanced integration technology takes metal oxide semiconductor field effect transistors (MOSFETs) as basic units and the MOSFET with planar gate can no longer effectively control the current due to the short channel effect, resulting in a three-dimensional Fin Field-Effect Transistor (Fin FET) [[1], [2], [3], [4]]. With the sharp reduction of the size of MOSFET devices, the channel length of MOSFET continues to approach and exceed the limits of lithography and other micromachining technologies. Therefore, people continue to develop new technologies to promote the extension of ‘Moore's law’ [5].Although in recent years, people have demonstrated integrated circuit devices and circuit prototypes based on new materials (such as carbon based CPU) and new principles (such as quantum computing) [6,7], it should be pointed out that CMOS circuits based on traditional Si based devices will still occupy the dominant position in the integrated circuit industry. It is the preferred scheme and mainstream trend in the direction of lower operating power consumption and richer function integration. New devices with enhanced functions can improve the integration of the ICs “softly” by realizing systems with multi functions and fewer basic cells. The field effect transistor with reconfiguration function (RFETs) aroused the attention of the academic community in recent years due to that its conduction type is reconfigurable during operation by changing the voltage of the program gate (PG) [[8], [9], [10]]. However, the scales involved in the reports on RFET is much larger than that achieved by today's mainstream FinFET, whether the reduced RFET can have the same performance as the mainstream technology is with uncertainty [[11], [12], [13], [14]]. RFETs generate carriers and form current by band to band tunneling (BTBT) phenomena near the interface between the source/drain electrodes and semiconductor, on which Schottky barriers for both conduction band and valence band are formed [15,16]. However, to realize the transistor with switchable conductivity type, two independent gate electrodes are required. However, the PG needs independently power supply, and the interconnection becomes more complicated. A single gate controlled RFET which simplifies the device structure of RFET is proposed [17]. However, the device is asymmetric, and the source/drain electrodes can not be exchanged as the mainstream CMOS technology. To achieve bidirectional function, in this work, we proposed a highly integrated nonvolatile bidirectional reconfigurable FET controlled by a single gate (SGCN-BRFET) is proposed. A floating program gate (FPG) formed on both source/drain sides is designed to achieve the nonvolatile function, the bidirectional function, and the reconfigurable function at the same time. Instead of the independently powered program gate (PG) of BRFET, the proposed SGCN-BRFET can be programmed by the CG itself. Thereafter, the interconnection can be simplified. The conduction type of the SGCN-BRFET is reconfigured by programming the FPG with different type of charges into the FPG. By optimizing the quantities of the stored charges, the FPG effective voltage can be changed to achieve higher forward current and lower leakage current. The principle of the proposed SGCN-RFET is interpreted through energy band theory. The device performance has been compared with BRFET. The influences of FPG to the performance of SGCN-BRFET have been analyzed.

## 2 Device structure

**Fig. 1:**
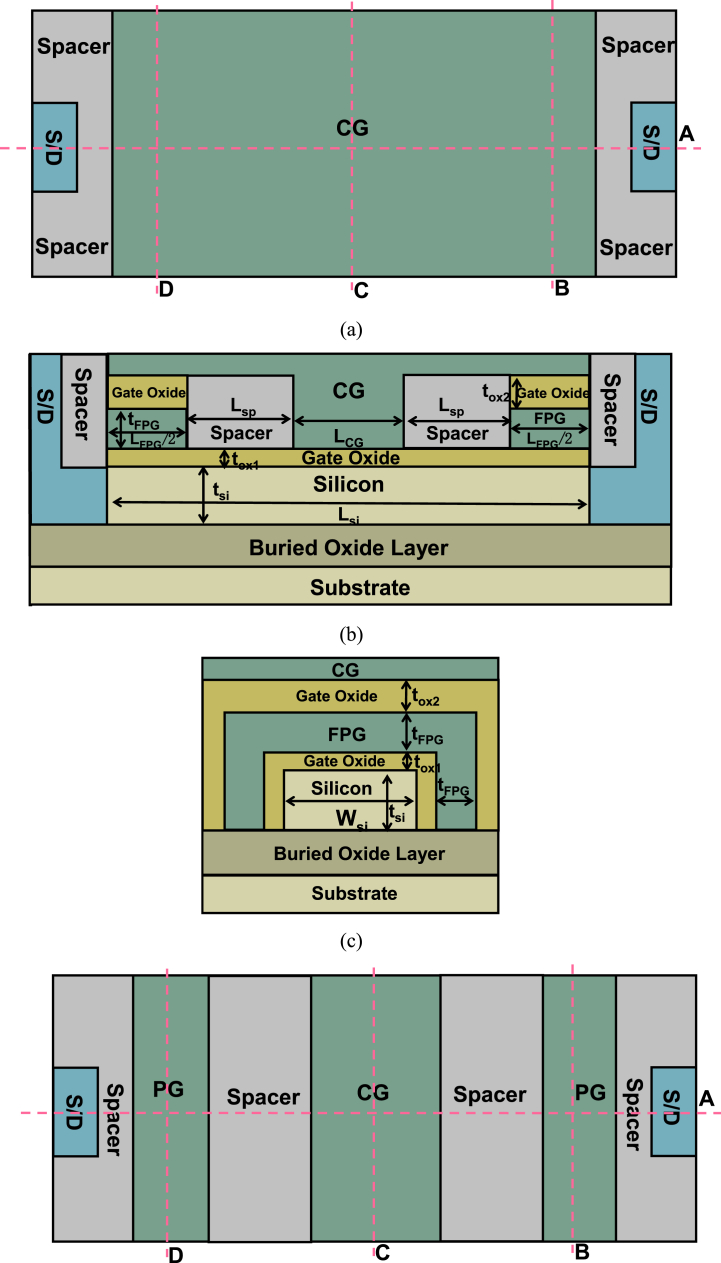

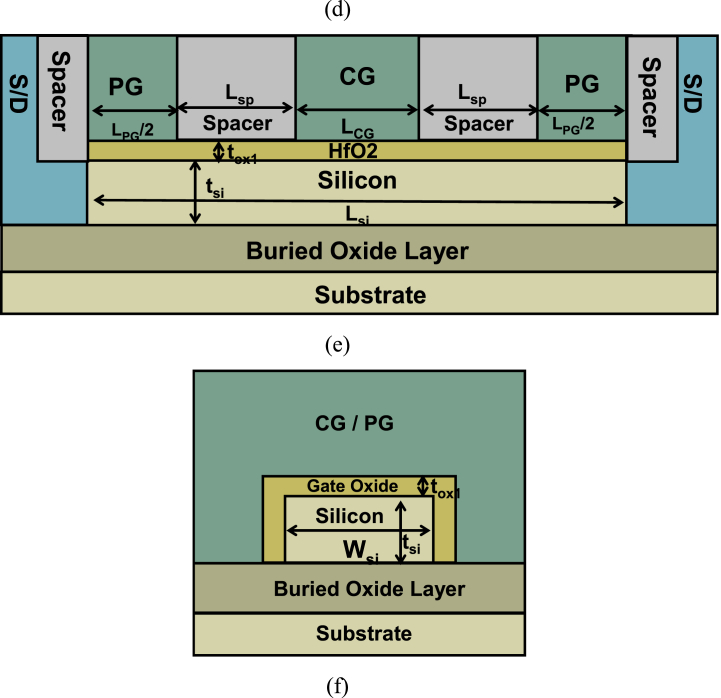
(a) Front view of SGCN-BRFET. (b) Cross view of SGCN-BRFET along cutting line A in Fig. 1 (a). (c) Cross section of SGCN-BRFET along cutting line B or D in Fig. 1 (a). (d) Front view of BRFET. (e) Cross view of BRFET along cutting line A in Fig. 1 (d). (f) Cross view of BRFET along cutting line B, C or D in Fig. 1 (d) or along cutting line C in Fig. 1 (a).

Fig. 1 (a) is a front view of SGCN-BRFET, Fig. 1 (b) is a cross view along cutting line A in Fig. 1 (a). Fig. 1 (c) is a cross view along cutting line B or D in Fig. 1 (a). The adopted parameters for SGCN-BRFET are shown in Table 1. Taking an n-type SGCN-BRFET as an example, when the drain-to-source voltage (V_DS_) is forwardly biased, if there is sufficient number of positive charges stored in FPG, the FPG is with an effective positive voltage and the strength of BTBT effect is controllable. Then the source/drain resistances induced by the Schottky junction is adjustable and the SGCN-BRFET is switchable. Fig. 1 (d) is a main view of the BRFET. Fig. 1 (e) is a cross view along the cutting line A in Fig. 1 (d), and Fig. 1 (f) is a cross view along cutting line B, C or D in Fig. 1 (d) or along cutting line C in Fig. 1 (a). Similar parameter labeling and parameter selection are used as much as possible to ensure the rationality of comparison. The parameter selection is also shown in Table 1.

**Table 1:** parameters selection for SGCN-BRFET.

Parameters	Values
the total length in silicon (L_si_);	20 nm
the length of the central part of CG (L_CG_);	5 nm
the half length of the FPG (L_FPG_/2);	5 nm
the total length of PG (L_PG_);	5 nm
the spacer length between FPG/PG and CG (L_sp_);	5 nm
The HfO_2_ thickness above silicon (t_ox1_);	0.7 nm
The HfO_2_ thickness between FPG and CG (t_ox2_);	1.4 nm
the silicon body thickness (t_si_);	5 nm
the total silicon body width (W_si_);	5 nm
the relative permittivity of the HfO_2_ layer (ε_HfO2_);	21.976
the relative permittivity of the Spacer (ε_spacer_);	3.89
the barrier height formed between the conduction band of silicon and the S/D electrodes. (qφ_bn_);	0.56eV
the barrier height formed between the valence band of silicon and the S/D electrodes (qφ_bp_);	0.52eV
The FPG charge (Q);	from 1.12 × 10^−17^C to 2.8 × 10^−17^C
the drain-to-source voltage (V_DS_)	−0.8V–0.8V
the gate-to-source voltage (V_GS_)	−0.8V–0.8V
the program gate voltage (V_PG_)	−0.8V–0.8V
the floating program gate voltage (V_FPG_)	−0.2V–1.2V

## 3 Results and discussions

**Fig. 2:**
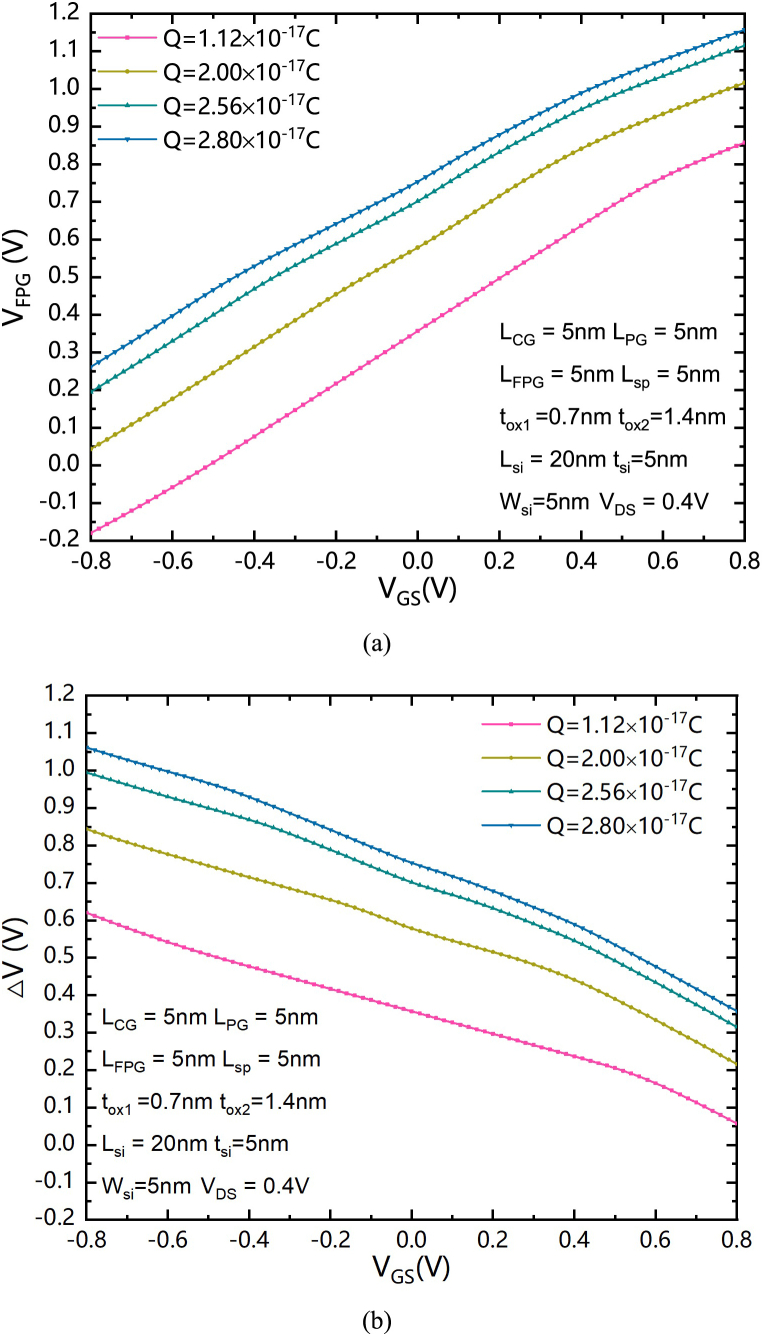
(a) the variation of V_FPG_ corresponding to Q and V_GS_. (b) The Variation of △V (△V=V_FPG_-V_GS_) corresponding to Q and V_GS_.

The performance of SGCN-BRFET has been investigated through TCAD simulations [18]. In this paper, models such as BTBT, Boltzman distribution, mobilities, band gap narrowing, and Auger recombination are all turned on to simulate the output characteristics. The variation of the V_FPG_ corresponding to Q and V_GS_ are shown in Fig. 2(a). When V_GS_ is fixed, V_FPG_ increases with the increase of Q. When Q is fixed, V_FPG_ shows obvious coupling effect with V_GS_, and is generally proportional to V_GS_. This makes V_FPG_ different from the V_PG_ of BRFET. It is no longer a fixed quantity, but a variable function that changes with the change of V_GS_. When the charge is written with a proper amount, V_FPG_ can achieve an effective value that higher than V_GS_ when CG is positively biased and can also decrease with the decreasing of V_GS_. When CG is reversely biased, V_FPG_ can be reduced to around 0V. For RFET, the way to generate carriers in the source region is by applying high voltage to PG, thus triggering BTBT effect to generate electron hole pairs. For SGCN-BRFET, due to this coupling effect, V_FPG_ is no longer constant, which makes the intensity of BTBT effect can be reduced under subthreshold or reverse biased conditions. Thus, the generation rate of electron hole pairs can be reduced, bringing the possibility of reducing static power consumption and reverse leakage current. Fig. 2(b) shows the between V_GS_ and the △V (△V equals to V_FPG_-V_GS_). △V is reduced with the increasing of V_GS_, while gradually increasing with the reducing of V_GS_. △V is much smaller than the difference between V_PG_ and V_GS_, due to the fixed V_PG_ of BRFET.

**Fig. 3:**
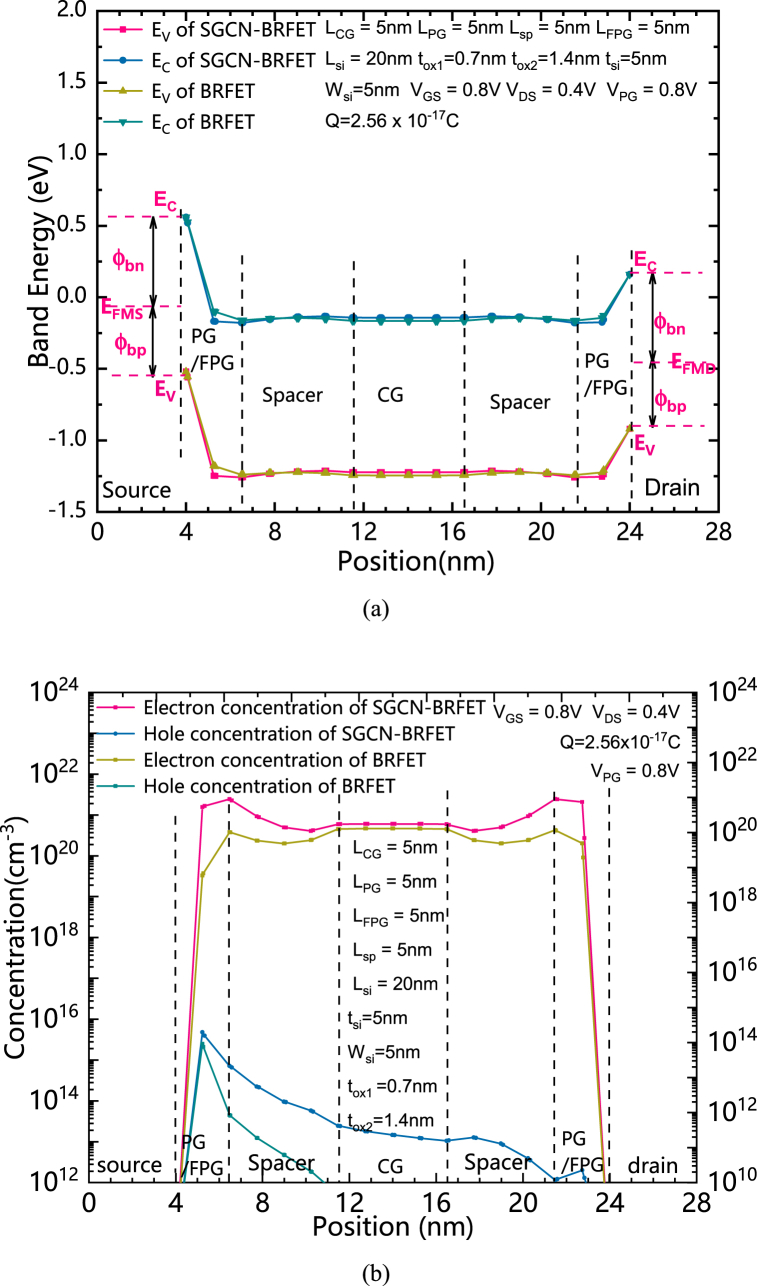
(a) the distributions of the bottom of the conduction band and the top of the valence band of both structures under forward biased conditions of n-type. (b) the distributions of both electron concentration and hole concentration of both structures under forward biased conditions of n-type.

The principle of the proposed SGCN-BRFET can be interpreted through the energy band theory. The distributions of the bottom of the conduction band and the top of the valence band of both structures under forward biased conditions of n-type are shown in Fig. 3 (a). The CG of both SGCN-BRFET and BRFET is forwardly biased. The FPG of SGCN-BRFET is written with a certain mount of Q. The PG of the BRFET is positively biased. As shown in Fig. 3 (a), E_FMS_ is the source Fermi level, while E_FMD_ is the drain Fermi level. E_C_ and E_V_ represent the bottom of the conduction band and the top of the valence band, respectively. The energy levels of both SGCN-BRFET and BRFET are pulled down in the entire silicon with the cooperation of CG and FPG/PG. Fig. 3 (b) shows the distributions of both electron concentration and hole concentration of both structures under forward biased conditions of n-type. Due to the tunnel effect is induced from energy band bending, an enormous number of electron-hole pairs are generated in the source regions of both structures under the condition of positively biased PG and positively charged FPG. The source electrode can accept the holes from the valence band (or, in fact, the source electrode provides electrons for the valence band to fill the holes of valence band) and for the conduction band, the electrons generated from tunnel effect in the source side can be dragged to the drain electrode by the positive V_DS_. And a large amount of forward conduction current can be formed. It is worth noting that the equivalent voltage in the FPG which is written with positive charge will increase with the increasing of V_GS_. It is different from the fixed V_PG_ of the BRFET, because the FPG will not produce a fixed series resistance to the device that limits the increase of the forward conduction current, so the forward current can continue to increase with the increasing of V_GS_.

**Fig. 4:**
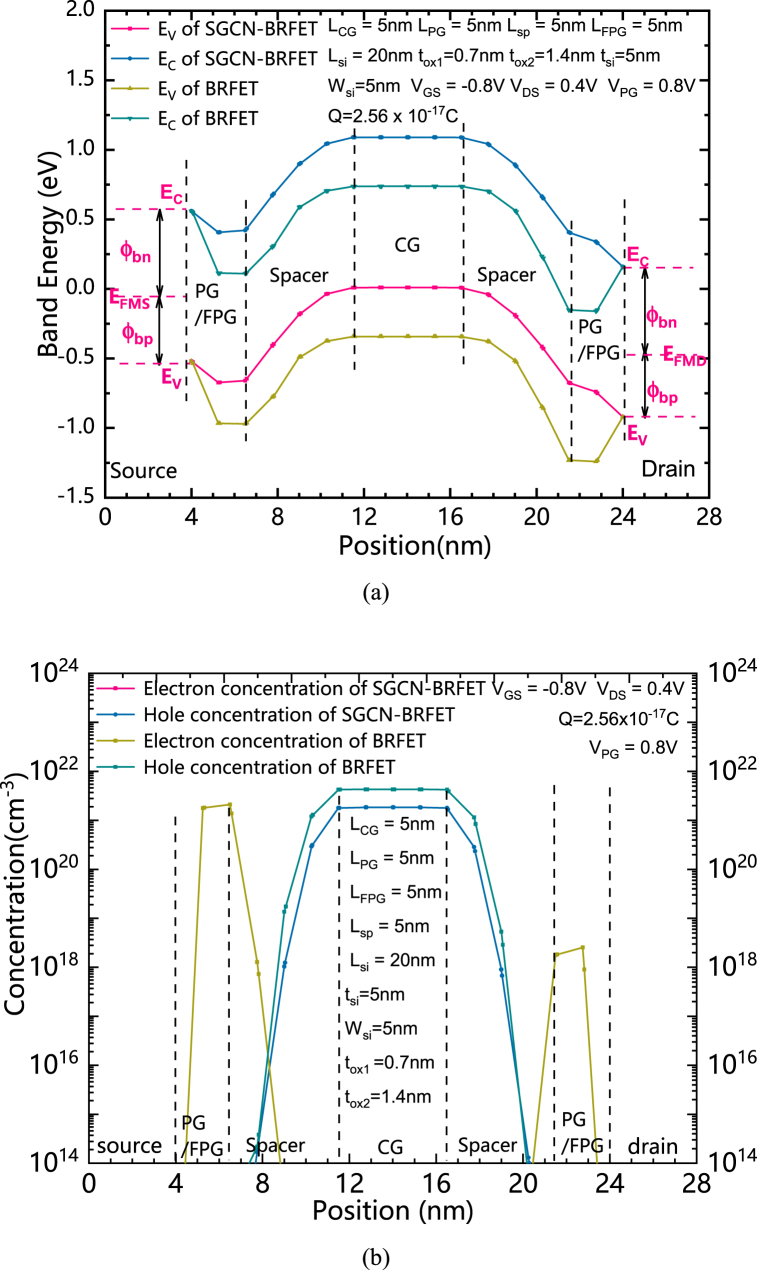
(a) the distributions of the bottom of the conduction band and the top of the valence band of both structures under reverse biased conditions of n-type. (b) The distributions of both electron and hole concentrations of both structures under reverse biased conditions of n-type.

The distributions of the bottom of the conduction band and the top of the valence band of both structures under reverse biased conditions of n-type are shown in Fig. 4 (a). The distributions of both electron and hole concentrations of both structures under reverse biased conditions of n-type are shown in Fig. 4 (b). The CG of both SGCN-BRFET and BRFET is reversely biased. The FPG of SGCN-BRFET is written with a mount of positive charge. The PG of the BRFET is positively biased. As shown in Fig. 4 (a), both the band energy of SGCN-BRFET and the band energy of BRFET in the central region of silicon are pulled up by CG. For the BRFET, the band energy on both sides under the control of PG are pull down. Therefore, a large band bending can be formed in the spacer regions between PG and CG. The only way to reduce the intensity of band bending is to prolong L_sp_. However, this is unfavorable for the improvement of integration. For SGCN-BRFET, unlike the BRFET, due to the coupling effect, V_FPG_ can be also decreased with the decreasing of V_GS_. Thus, the band energy in the region under the control of FPG can also be pull up to some extent. Thereafter, the electric field intensity in the silicon on both sides can be largely reduced. This leads to the weakening of the tunnel effect near the source region, thus preventing the generation of enormous number of electron hole pairs. As Fig. 4 (b) shows, comparing to the BRFET, the electron concentration generated by tunnel effect on both sides is largely reduced. Since the energy levels in the conduction band at the source side are almost empty, even if there is energy band bending in spacer region, there will not be a large number of BTBT effects, so that the holes in valence band located in the silicon region under the control of CG cannot be discharged through the source electrode. Therefore, a large amount of leakage current will not be generated.

**Fig. 5:**
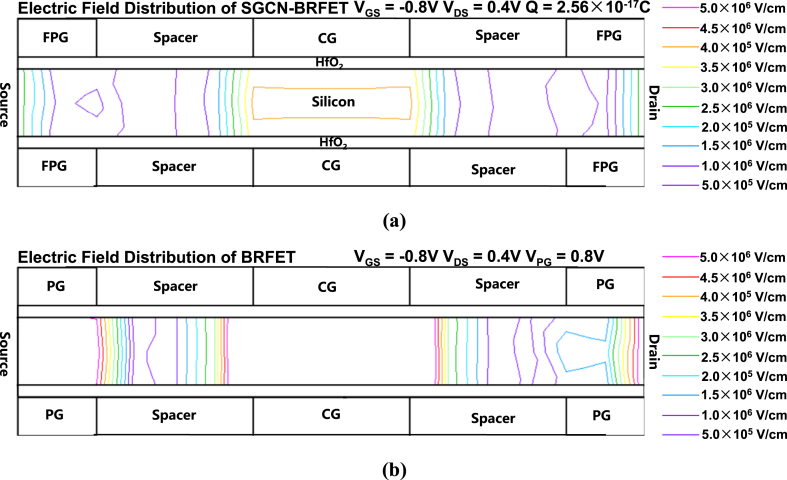
The intensity of electric field of (a) SGCN-BRFET and (b) BRFET in reverse state of n-type.

Fig. 5 (a) and Fig. 5 (b) show the intensity of electric field of SGCN-BRFET and BRFET in reverse state of n-type, respectively. The maximum electric field intensity in part off silicon region of BRFET corresponding to the spacer region reaches 5.0 × 10^6^ V/cm, much higher than that of SGCN-BRFET.

**Fig. 6:**
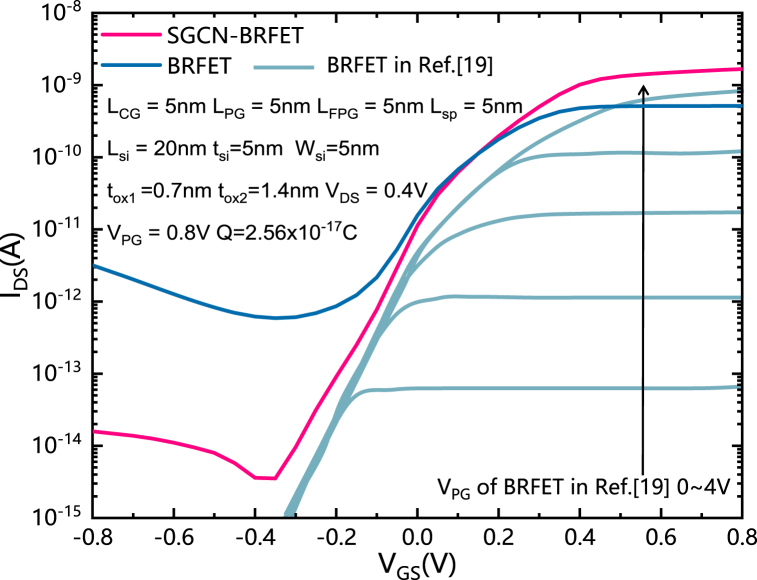
The I_DS_-V_GS_ characteristics of the SGCN-BRFET, the BRFET and the normalized experimental BRFET in Ref. [19].

The I_DS_-V_GS_ characteristics of the SGCN-BRFET, the BRFET and the normalized experimental BRFET in Ref. [19] are shown in Fig. 6. Compared with BRFET, SGCN-BRFET achieves increased on state current and decreased reverse current simultaneously. From the perspective of reverse state, the leakage current of SGCN-BRFET is nearly two orders of magnitude lower than BRFET. The comparison results of Fig. 6 are consistent with the analysis results in Fig. 3, Fig. 4.

**Fig. 7:**
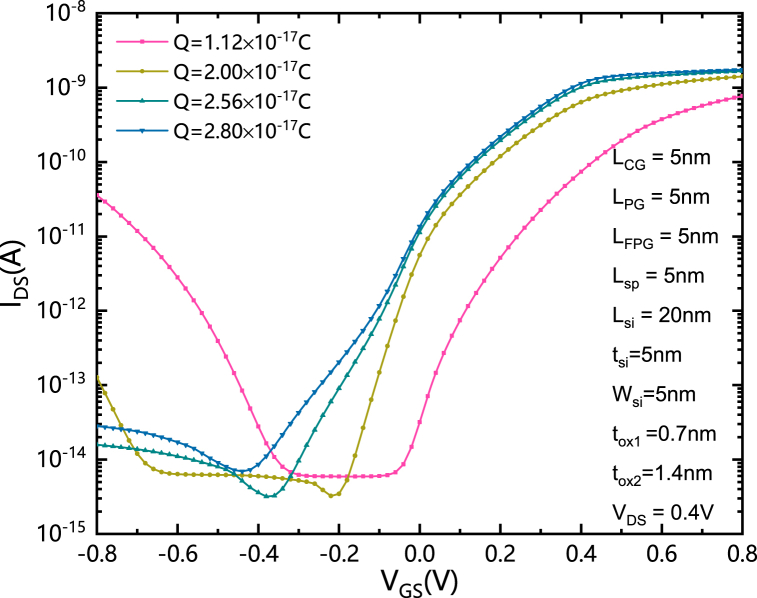
The transfer characteristics of SGCN-BRFET with different Qs.

The dependence between I_DS_-V_GS_ characteristics and Qs is shown in Fig. 7. In order to determine the conduction mode and avoid the insufficient band bending near the source side due to low effective voltage of the FPG, the FPG should be programmed with enough charges. For Q equals to 1.12 × 10^−17^C, the forward current is limited. Based on the analysis of Figs. 2–5, it is easy to know that this is caused by the insufficient Q and the corresponding low V_FPG_. At the same time, the insufficient Q will also cause the V_FPG_ to be too low when the gate is reversely biased, which will increase the strong band bending near the interface between silicon and the source electrode or drain electrode, as well as the reverse current. The increased Q helps to improve the V_FPG_ in the reverse state. However, it should be noted that the amount of charge needs to be optimized, because excessive charge will cause the V_FPG_ to be too high, thus increasing the intensity of strongest electric field in silicon and the corresponding increase of the reverse leakage current. The optimize value of Q for the selected structural parameters is about 2.56 × 10^−17^C.

**Fig. 8:**
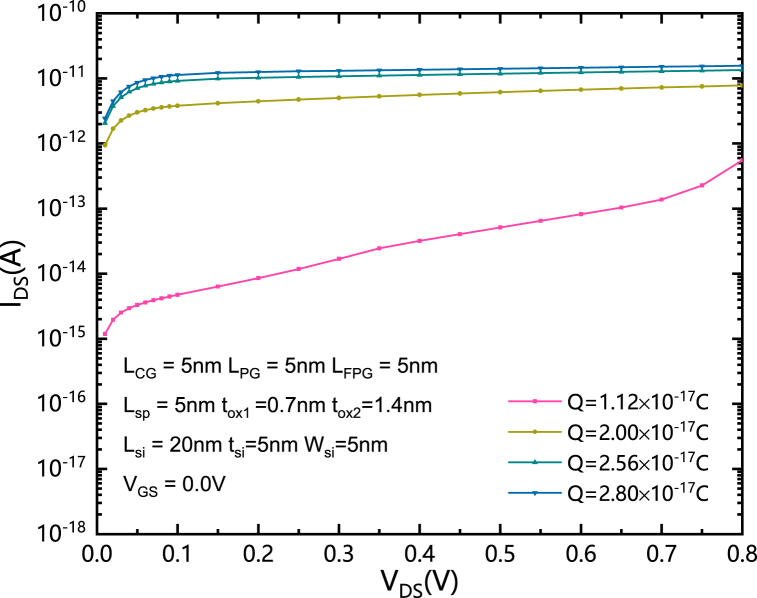
The relationship between I_DS_ and V_DS_ in the subthreshold region at V_GS_ equals to 0V.

Fig. 8 shows the relationship between I_DS_ and V_DS_ in the subthreshold region at V_GS_ equals to 0V. It can be seen that when FPG is fully charged, the magnitude of the subthreshold current almost does not change with the change of V_DS_, while when the FPG is not fully charged, the subthreshold current becomes smaller than the sufficient charged case at the same V_GS_ due to its lower effective voltage and the weakening of its control ability to the band bending. And in this case, the influence of V_DS_ to the band bending becomes more obvious comparing to that of FPG. In other words, when the amount of Q becomes low, the degree of band bending on the drain side has changed from being dominated by the FPG to being dominated by V_DS_.

**Fig. 9:**
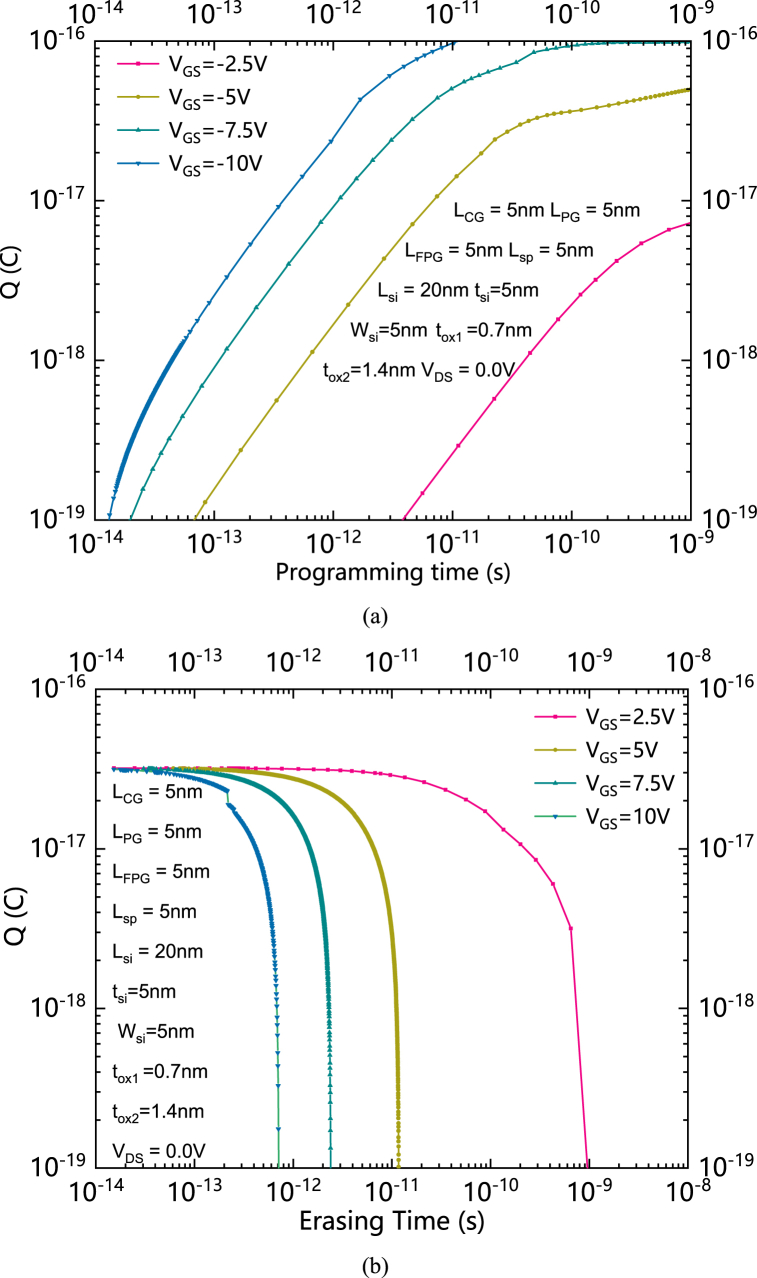
(a) the relationship between Q and the programming time with negative V_GS_ s (b) The relationship between Q and erasing time with positive V_GS_s.

The relationship between Q and the programming time with negative V_GS_ s is shown in Fig. 9 (a). The relationship between Q and erasing time with positive V_GS_s is shown in Fig. 9 (b). During the program operation, the source and drain electrodes are both grounded, and a large gate voltage is applied. The Q is increasing with the increasing of the time of the program operation, and the time of the program operation can be saved by increasing V_GS_. Positive charges can be stored by applying a large negative V_GS_. Then the SGCN-BRFET works as an N-type device. The positive charges are eliminated by increasing V_GS_. The erase operation can be speed up by increasing V_GS_.

## 4 Conclusions

In this paper, a highly integrated nonvolatile bidirectional reconfigurable FET controlled by a single gate is proposed. A floating program gate (FPG) formed on both source/drain sides is designed to achieve the nonvolatile function, the reconfiguration function, and the bidirectional function at the same time. Instead of the independently powered program gate (PG) of BRFET, the proposed SGCN-BRFET can be programmed by the CG itself. Thereafter, the interconnection can be simplified. The SGCN-BRFET is reconfigured by programming the FPG with different type of charges. By optimizing the quantities of the stored charges, the FPG effective voltage can be changed to achieve higher forward current and lower leakage current. The physical mechanism of the proposed SGCN-RFET has been systematically analyzed. The device performance has been compared with BRFET. The influence of the amount of charge to the device performance has also been discussed in detail. The V_FPG_ increases with the increase of Q and shows obvious coupling effect with V_GS._ V_FPG_ can achieve an effective value that higher than V_GS_ when CG is positively biased and can also decrease with the decreasing of V_GS_. It makes the intensity of BTBT effect can be reduced under subthreshold or reverse biased conditions and the maximum electric field intensity of SGCN-BRFET in reverse biased state is much lower than that of BRFET. The principle of the SGCN-BRFET is also interpreted through the energy band theory. The optimize value of Q for the selected structural parameters is about 2.56 × 10^−17^C. When FPG is fully charged, the magnitude of the subthreshold current almost does not change with the change of V_DS_, while when the FPG is not fully charged, the subthreshold current becomes smaller than the sufficient charged case at the same V_GS_ due to its lower effective voltage and the weakening of its control ability to the band bending.

## Author contribution statement

Xiaoshi Jin; Xi Liu, Shouqiang Zhang & Xiaoshi Jin: Conceived and designed the experiments; Performed the experiments; Analyzed and interpreted the data; Contributed reagents, materials, analysis tools or data; Wrote the paper.

1) conceived and designed the experiments;

2) performed the experiments;

3) analyzed and interpreted the data;

4) contributed reagents, materials, analysis tools or data;

5) wrote the paper.

## Data availability statement

Data will be made available on request.

## Declaration of competing interest

The authors declare that they have no known competing financial interests or personal relationships that could have appeared to influence the work reported in this paper.
